# Draft Genome Sequence of a 16SrII-A Subgroup Phytoplasma Associated with Purple Coneflower (*Echinacea purpurea*) Witches’ Broom Disease in Taiwan

**DOI:** 10.1128/genomeA.01398-15

**Published:** 2015-11-25

**Authors:** Shu-Heng Chang, Shu-Ting Cho, Chung-Li Chen, Jun-Yi Yang, Chih-Horng Kuo

**Affiliations:** a Institute of Biochemistry, National Chung Hsing University, Taichung, Taiwan; b Institute of Plant and Microbial Biology, Academia Sinica, Taipei, Taiwan; c Department of Agronomy, National Chung Hsing University, Taichung, Taiwan; d Molecular and Biological Agricultural Sciences Program, Taiwan International Graduate Program, National Chung Hsing University and Academia Sinica, Taipei, Taiwan; e Biotechnology Center, National Chung Hsing University, Taichung, Taiwan

## Abstract

The bacterial genus “*Candidatus* Phytoplasma” contains a group of insect-transmitted plant pathogens in the class *Mollicutes*. Here, we report a draft genome assembly and annotation of strain NCHU2014, which belongs to the 16SrII-A subgroup within this genus and is associated with purple coneflower witches’ broom disease in Taiwan.

## GENOME ANNOUNCEMENT

Phytoplasmas are a group of phloem-limited plant pathogens vectored by sap-feeding insects (1). Because of the difficulties involved in cultivating these bacteria outside of their hosts, genome sequencing and comparative analysis have been adopted as a major tool to study them (2, 3). The strain NCHU2014 was associated with purple coneflower (*Echinacea purpurea*) witches’ broom disease in Taiwan (4). Based on its 16S rRNA gene sequence, this strain is most closely related to “*Candidatus* Phytoplasma australasiae” and has been assigned to the 16SrII-A subgroup within the genus. To facilitate future investigation on the biology of this bacterium, as well as to improve the taxon sampling of available phytoplasma sequences for comparative analyses, we report a draft genome assembly of this bacterium here.

The bacterial strain NCHU2014 was collected from naturally infected purple coneflower at the agricultural experiment station of National Chung Hsing University (Taichung, Taiwan) in June 2014. Subsequently, the strain was transferred to periwinkle (*Catharanthus roseus*) through dodder (*Cuscuta australis*) and maintained by grafting. Mature leaves from artificially infected periwinkle were collected for total DNA extraction by using the DNeasy plant minikit (Qiagen). The Illumina MiSeq platform was used to generate 300-bp reads from one paired-end library (~500 bp insert, 34,321,466 reads).

The procedures for genome assembly and annotation were based on those described in our previous studies (5, 6). Briefly, the initial *de novo* assembly was performed using Velvet version 1.2.10 (7). Putative phytoplasma contigs were identified by running BLASTx (8) searches against the NCBI nonredundant database (9). Additionally, the contigs were checked against the periwinkle chloroplast genome (10) to exclude possible contamination from the plant host. The draft genome of a closely related phytoplasma associated with peanut witches’ broom (PnWB) disease (5) was used as a reference for scaffolding. PCR and Sanger sequencing were used for gap filling. For final verification, the Illumina raw reads were mapped to the assembly using BWA version 0.7.12 (11), programmatically checked using the mpileup program in SAMtools package version 1.2 (12), and visually inspected using IGV version 2.3.57 (13).

The programs RNAmmer (14), tRNAscan-SE (15), and Prodigal (16) were used for gene prediction. The gene names and product descriptions were first annotated based on the homologous genes in the PnWB phytoplasma (5) and aster yellows phytoplasma (17), as identified by OrthoMCL (18). Subsequent manual curation was based on BLASTp (8) searches against the NCBI nonredundant database (9) and the KEGG database (19, 20).

The first version of this draft genome contains 28 contigs with a combined size of 545,427 bp; the average G+C content is 23.9%. The annotation includes four rRNA genes, 26 tRNA genes, and 433 protein-coding genes.

### Nucleotide sequence accession numbers.

This whole-genome shotgun project has been deposited at DDBJ/EMBL/GenBank under the accession number LKAC00000000. The version described in this paper is the first version, LKAC01000000.
